# Poly(2-Hydroxyethyl Methacrylate) Hydrogel-Based Microneedles for Bioactive Release

**DOI:** 10.3390/bioengineering11070649

**Published:** 2024-06-25

**Authors:** Manoj B. Sharma, Hend A. M. Abdelmohsen, Özlem Kap, Volkan Kilic, Nesrin Horzum, David Cheneler, John G. Hardy

**Affiliations:** 1 Department of Chemistry, Lancaster University, Lancaster LA1 4YB, UK; 2 School of Engineering, Lancaster University, Lancaster LA1 4YW, UK; 3 Department of Pharmaceutics and Industrial Pharmacy, Ain Shams University, African Union Organization Street, Abbassia, Cairo 11566, Egypt; 4 Department of Engineering Sciences, Izmir Katip Celebi University, Izmir 35620, Türkiyenesrin.horzum.polat@ikc.edu.tr (N.H.); 5 Department of Electrical and Electronics Engineering, Izmir Katip Celebi University, Izmir 35620, Türkiye; volkan.kilic@ikc.edu.tr

**Keywords:** hydrogels, microneedles, drug delivery, estradiol, melatonin, meropenem

## Abstract

Microneedle arrays are minimally invasive devices that have been extensively investigated for the transdermal/intradermal delivery of drugs/bioactives. Here, we demonstrate the release of bioactive molecules (estradiol, melatonin and meropenem) from poly(2-hydroxyethyl methacrylate), pHEMA, hydrogel-based microneedle patches in vitro. The pHEMA hydrogel microneedles had mechanical properties that were sufficiently robust to penetrate soft tissues (exemplified here by phantom tissues). The bioactive release from the pHEMA hydrogel-based microneedles was fitted to various models (e.g., zero order, first order, second order). Such pHEMA microneedles have potential application in the transdermal delivery of bioactives (exemplified here by estradiol, melatonin and meropenem) for the treatment of various conditions.

## 1. Introduction

Microneedles have emerged as an enabling technology for the delivery of bioactive molecules, etc. [1,2]. Microneedles can be produced from a variety of materials (including metals, ceramics, polymers, etc.) via a variety of techniques, yielding microneedles with defined structures and properties [3,4,5,6,7,8,9,10]. Soft lithography techniques are used to fabricate/replicate structures using elastomeric masks/molds/stamps [11,12]. This has proven popular in the production of microneedles, most often employing the commercially available elastomer polydimethylsiloxane (PDMS) [13,14].

Many techniques exist by which to manufacture microneedles, including (but not limited to) additive manufacturing [15,16], molding [7,17], and laser ablation [9,11,12,13,18], as reviewed extensively [13,19,20,21]. Soft lithography encompasses methods involving elastomeric molds or stamps to replicate structures with high fidelity [15,16], and PDMS is routinely used in soft lithography due to its mechanical properties, ease of fabrication, and inexpensive nature, which makes it accessible for researchers worldwide [22]. Consequently, soft lithography techniques are often used for microneedle fabrication due to their versatility, precision, and cost-effectiveness compared to alternative methods [17].

We select melatonin, meropenem, and estradiol as representative drugs to showcase the versatility of microneedle arrays across a spectrum of therapeutic applications. Each chosen drug addresses a distinct medical domain, contributing to the exploration of microneedle capabilities. First, melatonin, a neurohormone, is recognized for its role in regulating circadian rhythms and sleep–wake cycles [23]. By employing microneedle technology for melatonin delivery, our study extends its focus beyond conventional administration methods. The microneedle-mediated delivery of melatonin holds promise for targeted neuroprotection, offering a potential avenue for addressing sleep disorders, neurological conditions, and other related medical challenges. Meropenem is a broad-spectrum antibiotic [24], and the localized and controlled release facilitated by microneedles could enhance the efficacy of meropenem, presenting a novel approach to combatting bacterial infections with reduced systemic side effects. Estradiol, a key estrogen hormone, is central to our exploration of hormone replacement therapies [25]. Microneedle technology offers a unique advantage in delivering hormones like estradiol, providing a minimally invasive and controlled means of hormone administration. This application holds promise for managing conditions related to hormonal imbalances, particularly in the context of women’s health.

Medical model tissues (“phantom tissues”) designed to replicate the characteristics of healthy/unhealthy tissues can be used for the development of biomaterials, medical devices, computational models, algorithms, surgical planning, etc. [26,27,28,29,30,31]. Such phantom tissues are particularly useful for research and development due to the lack of freely available tissues from humans/animals for human/veterinary medicine [32,33]. Phantom tissues are often composite materials (comprising natural/synthetic polymers, inorganic components [salts, etc.], and mixtures of oil and water), and the manufacturing method used to produce such phantom tissues is dependent on the specific tissues being mimicked and specific experiments being undertaken, aspects of which are covered in excellent reviews [34,35,36,37,38]. Using such tissue models helps bridge the gap between in vitro and in vivo studies, providing a reproducible environment for investigations into microneedle–tissue interactions and drug-release dynamics, and offering insights into the performance of microneedle-based drug delivery systems [39,40,41].

We have previously employed PDMS templates for the development of poly(2-hydroxyethyl methacrylate) (pHEMA) hydrogel-based microneedles with various architectures and demonstrated their potential application for the transdermal delivery of drugs in vitro (specifically metformin, owing to its potential for the treatment of ageing, cancer, diabetes, etc.) [42]. We utilized microneedle array master templates designed via computer-aided design (CAD), fabricated array master templates (composed of light-curing methacrylic/acrylic resin, Envision TECH HTM 140 V2) using 3D stereolithography, sprayed the master templates with release liner, and filled them with a degassed polydimethylsiloxane (PDMS) precursor mixture (a SYLGARD^®^ 184 PDMS kit, Dow Inc., Midland, MI, USA) that was baked to crosslink the PDMS, followed by cooling. The PDMS templates were filled with hydrogel precursors and baked to produce crosslinked hydrogel-based microneedle arrays, after which they were cooled and washed to remove any contaminants. Of the nine different microneedle array designs, we observed the most reliable microneedle array production from the PDMS microneedle array template with triangle/pyramid structures and used that in all further studies (employing pHEMA hydrogels derived from baking hydrogel precursors (2-hydroxyethyl methacrylate (HEMA), poly(ethylene glycol)dimethacrylate (PEGDMA, average Mn 550) and benzoyl peroxide (BPO)). Confocal microscopy showed the length of the pHEMA hydrogel microneedles produced was approximately 238 ± 97 μm.

To further demonstrate the versatility of this approach to generating microneedle-based biomedical microdevices, here we demonstrate their interfacing with phantom tissues mimicking normal healthy and cancerous tissues [32], and efficacy in delivering other bioactive molecules (specifically, estradiol [a hormone] [43,44,45], melatonin [a hormone] [46,47], and meropenem [a broad-spectrum antibiotic] [48,49]) in vitro.

## 2. Materials and Methods

### 2.1. Preparation of PDMS Microneedle Templates

Microneedle templates were produced as previously described [42]. The 3D-printed templates were placed on a ceramic tile, sprayed with release liner (Ambersil Silicone Mould Release Agent Plastic, RS Components UK, Corby, UK), and allowed to dry. These templates were used to create microneedle molds using a SYLGARD^®^ 184 PDMS kit (Dow Inc., Midland, MI, USA; the purity of the silicone elastomer and curing agent was ≥99.5%). A 10:1 mixture of silicone elastomer to silicon elastomer curing agent was stirred in a plastic container and degassed in a vacuum desiccator until bubbles stopped rising to the surface. The templates were filled with the PDMS mixture (approximately 3 mL of the PDMS mixture was needed to fill them) and baked in an oven at 60 °C for 16 h, after which they were cooled, removed from the templates using spatulas and stored in plastic containers until use. Unless otherwise noted, everything was purchased from Sigma-Aldrich (Gillingham, UK) and used as supplied (the purity of all chemicals was ≥99%).

### 2.2. Preparation of pHEMA Microneedle Arrays

Microneedle arrays were produced as previously described [42]. Amounts of 20 mL of 2-hydroxyethyl methacrylate (HEMA), 0.2 mL of poly(ethylene glycol)dimethacrylate (PEGDMA, average Mn 550) and 88 mg of benzoyl peroxide (BPO) were mixed until homogeneous, then degassed in a vacuum desiccator, followed by the transfer of ca. 3 mL of the formulation to the microneedle mold. The samples were heated at 100 °C in an oven for 3 h, after which they were cooled to room temperature and thoroughly washed with deionized water over a period of a week to remove any non-crosslinked components (e.g., initiators, monomers, oligomers, etc.). Unless otherwise noted, everything was purchased from Sigma-Aldrich (Gillingham, UK) and used as supplied (the purity of all chemicals was ≥99%).

### 2.3. Preparation of Healthy and Cancerous Breast Phantom Tissues

#### 2.3.1. Preparation of Healthy Breast Phantom Tissues

Healthy breast phantom tissue was prepared by an adaptation of the literature [50]. In short, 0.2 g of *p*-toluic acid was added to 10 mL of *n*-propanol in a vessel, followed by heating to ≈90 °C and stirring until complete dissolution. This solution was added to 30 mL of deionized water while stirring, and 5 g of gelatin derived from porcine skin (50–100 kDa) was added. The beaker was covered with a plastic film to minimize water evaporation, and the mixture was heated on a magnetic hotplate stirrer until the solution became clear. Heating ceased once the solution turned clear, and the sample was allowed to cool to ≈65 °C, after which 33.6 mL of Mrs. Meyer’s clean day liquid surfactant (supplied by Amazon, Seattle, WA, USA) was added.

In parallel, 60 mL of paraffin oil was heated to 65 °C and then added into the solution, followed by the careful addition of 0.32 mL of formaldehyde solution, resulting in the formation of an emulsion characterized by a uniform, pale liquid appearance. The stirring continued until the temperature dropped below 50 °C. Finally, the solution was poured into a container (glass Petri dish with a diameter of 75 mm) and left to polymerize for about 24 h at room temperature (≈25 °C).

Unless otherwise noted, everything was purchased from Sigma-Aldrich (Gillingham, UK) and used as supplied (the purity of all chemicals was ≥99%). As a commercial product, the precise composition of Mrs. Meyer’s clean day liquid surfactant is a trade secret; however, the constituent ingredients are listed as follows: water, sodium lauryl sulfate, lauryl glucoside, lauramine oxide, polysorbate 20, glycerin, Ocimum basilicum (Basil) oil, Carum petroselinum (parsley) seed oil, Piper nigrum (black pepper) seed oil, Quillaja saponaria (soap) bark extract, fragrance, Aloe barbadensis leaf, tetrasodium glutamate diacetate, citric acid, PEG-5 cocoate, methylisothiazolinone, benzisothiazolinone.

#### 2.3.2. Preparation of Cancerous Breast Phantom Tissues

Cancerous breast phantom tissue was prepared by an adaptation of the literature [50]. In short, 0.2 g of *p*-toluic acid was added to 10 mL of *n*-propanol in a vessel, followed by heating to ≈90 °C and stirring until complete dissolution. This solution was added to 30 mL of deionized water while stirring, and 5 g of gelatin derived from porcine skin was added. The beaker was covered with a plastic film to minimize water evaporation, and the mixture was heated on a magnetic hotplate stirrer until the solution became clear. Heating ceased once the solution turned clear, and the sample was allowed to cool to ≈65 °C, after which 5.6 mL of Mrs. Meyer’s clean day liquid surfactant (supplied by Amazon, Seattle, WA, USA) was added.

In parallel, 10 mL of paraffin oil was heated to 65 °C and then added into the solution, followed by the careful addition of 0.32 mL of formaldehyde solution, resulting in the formation of an emulsion characterized by a uniform, pale liquid appearance. The stirring continued until the temperature dropped below 50 °C. Finally, the solution was poured into a container (glass Petri dish with a diameter of 75 mm) and left to polymerize for about 24 h at room temperature (≈25 °C).

Unless otherwise noted, everything was purchased from Sigma-Aldrich (Gillingham, UK) and used as supplied (the purity of all chemicals was ≥99%). As a commercial product, the precise composition of Mrs. Meyer’s clean day liquid surfactant is a trade secret, and the constituent ingredients are listed above.

### 2.4. Swelling Behavior of Phantom Tissues

To quantitatively assess the swelling behavior in phosphate-buffered saline (PBS, at pH 7.4, chosen to mimic the pH of the blood into which the microneedles would release the drugs if applied to healthy breast tissue), the percentage increase in dimensions was calculated for each phantom tissue. The formula for the percentage increase (*PI*) in each dimension (*D*) is expressed by Equation (1):

(1) $$PI=\left(\frac{D_{Final}-D_{Initial}}{D_{Initial}}\right)*100$$

The average swelling rate for each dimension was determined by calculating the mean of the percentage increase values. Furthermore, the standard deviation was computed to assess the variability in the swelling responses, utilizing Equation (2):

(2) $$\frac{\sqrt{\sum (x_{i}-\bar{x})2}}{N}$$

where *x_i_* represents each individual data point, $\bar{x}$ is the mean, and *N* is the number of data points. These statistical measures provided a comprehensive understanding of the swelling characteristics, enabling meaningful comparisons and insights into the heterogeneity or homogeneity within each set of data. The resulting statistical analyses contribute essential information to the refinement of microneedle drug delivery systems, emphasizing the importance of robust and reproducible tissue-mimicking models.

### 2.5. Microneedle Modulus Estimation

Controlled forces were incrementally applied using an Instron 3345 Universal Testing Machine (Wycombe, UK), while load and displacement were recorded at 500 Hz, respectively. A microneedle can be considered to be a columnar structure with a varying cross-section. Whilst under compression, for small strains, the stress in the microneedle can be defined by Hooke’s law (3):

(3) $$\sigma \left(x\right)=E\varepsilon \left(x\right)$$

where $\sigma \left(x\right)$ is the stress as a function of the distance x along the length of the microneedle, ε is the strain and E is the effective elastic modulus. Let the applied force be F and the cross-sectional area of the microneedle along its length be $A\left(x\right)$; then, the stress becomes $\sigma \left(x\right)=F/A\left(x\right)$. The strain is defined as $\varepsilon \left(x\right)=du/dx$, where u is the deformation of the microneedle. The deformation of a microneedle (of height h) under compression can therefore be defined as (4):

(4) $$u=\frac{F}{E}\int_{0}^{h}\frac{dx}{A\left(x\right)}$$

If the microneedle has a geometry defined by a truncated cone with a linearly varying radius, with base radius $l_{b}$ and tip radius $l_{t}$, the cross-sectional area is (5):

(5) $$A\left(x\right)=\pi {\left[l_{t}+\frac{x}{h}\left(l_{b}-l_{t}\right)\right]}^{2}$$

A microneedle defined by a square cross-section with a linearly varying side length, with base side length $l_{b}$ and tip side length $l_{t}$, has a cross-sectional area of (6):

(6) $$A\left(x\right)={\left[l_{t}+\frac{x}{h}\left(l_{b}-l_{t}\right)\right]}^{2}$$

A microneedle defined by an equilateral triangular cross-section with a linearly varying side length, with base side length $l_{b}$ and tip side length $l_{t}$, has a cross-sectional area of (7):

(7) $$A\left(x\right)=\frac{\sqrt{3}}{4}{\left[l_{t}+\frac{x}{h}\left(l_{b}-l_{t}\right)\right]}^{2}$$

A microneedle defined by a pentagon cross-section with a linearly varying side length, with base side length $l_{b}$ and tip side length $l_{t}$, has a cross-sectional area of (8):

(8) $$A\left(x\right)=\frac{1}{4}\sqrt{5\left(5+2\sqrt{5}\right)}{\left[l_{t}+\frac{x}{h}\left(l_{b}-l_{t}\right)\right]}^{2}$$

And so on. Therefore, if the microneedle is defined by a geometry whereby the cross-sectional shape is a regular polygon, or circle, where only the characteristic length changes, and that in a linear fashion, the deformation can be calculated as (9):

(9) $$u=\frac{F}{CE}\int_{0}^{h}\frac{dx}{{\left[l_{t}+\frac{x}{h}\left(l_{b}-l_{t}\right)\right]}^{2}}$$

where C equals 1 for a square cross-section, π for a circle,$\;\frac{\sqrt{3}}{4}$ for an equilateral triangle, $\frac{1}{4}\sqrt{5\left(5+2\sqrt{5}\right)}$ for a pentagon, and so on. The solution of Equation (9) is (10):

(10) $$u=\frac{Fh}{CEl_{b}l_{t}}$$

If the microneedle is formed from a tip (of height $h_{t}$ and characterized by a linearly varying area) on a base prism of height $h_{b}$ and constant cross-sectional area A, then the deformation of the microneedle will be (11):

(11) $$u=\frac{F}{CE}\int_{0}^{h_{t}}\frac{dx}{{\left[l_{t}+\frac{x}{h_{t}}\left(l_{b}-l_{t}\right)\right]}^{2}}+\frac{Fh_{b}}{EA}$$

or (12):

(12) $$u=\frac{Fh_{t}}{CEl_{b}l_{t}}+\frac{Fh_{b}}{EA}=\frac{F}{E}\left(\frac{h_{t}}{Cl_{b}l_{t}}+\frac{h_{b}}{A}\right)$$

This predicts that for a microneedle with a linearly changing or constant cross-sectional area, the deformation is a linear function of the applied force. If a straight line is fitted to the force–displacement data, the gradient or stiffness, k, should be (13):

(13) $$k=\frac{E}{\left(\frac{h_{t}}{Cl_{b}l_{t}}+\frac{h_{b}}{A}\right)}$$

The stiffness of an array of n identical microneedles being compressed simultaneously would be (14):

(14) $$k=\frac{nE}{\left(\frac{h_{t}}{Cl_{b}l_{t}}+\frac{h_{b}}{A}\right)}$$

For the triangle pyramid structures adopted in this paper, Equation (14) reduces to (15):

(15) $$k=\frac{\sqrt{3}}{4}\frac{nl_{b}l_{t}E}{h_{t}}$$

Therefore, for a given stiffness, the effective elastic modulus can be calculated as (16):

(16) $$E=\frac{4}{\sqrt{3}}\frac{kh_{t}}{nl_{b}l_{t}}$$

where n = 100, $h_{t}$ = 343.2 µm, $l_{b}$ = 387.2 µm and $l_{t}$ = 7.5 µm.

### 2.6. Microneedle Insertion Study in Phantom Tissues

#### 2.6.1. Penetration Force Measurement

The pHEMA microneedle arrays (11 × 11) were swelled in PBS for 24 h. The microneedle penetration force measurement procedure involves pressing the microneedles onto the phantom tissues mimicking normal and cancerous breast tissue with a length of 1.1 cm and a thickness of 1.2 cm, respectively. After securely positioning the PBS swollen microneedle arrays perpendicularly above the tissue phantoms, on a stable platform, the force measurement system was calibrated. A preload of 0.1 N was applied, and an indentation rate of 0.1 mm/s was maintained. Controlled forces were incrementally applied using an Instron 3345 Universal Testing Machine (Wycombe, UK), while load and displacement were recorded at 500 Hz, respectively. All the experiments were completed in triplicate (*n* = 3), and data are reported as the mean average ± standard deviation.

#### 2.6.2. Confocal Imaging

The pHEMA microneedle arrays (11 × 11) were swelled in PBS for 24 h. The arrays were positioned above the phantom tissues and an external pressure of 14 N was applied to facilitate the insertion of the microneedles into the phantom tissues, followed by their removal from the phantom tissue and imaging with confocal microscopy (using a LEXT OLS5000 3D measuring laser microscope, images were taken with the MPLFLN10x LEXT objective lens with a 1× zoom in 3D standard mode; Olympus, Evident Europe GmbH, Stansted, UK). The penetration efficiency was calculated by dividing the total number of needles in the microneedle array by the number of needle array marks on the phantom tissue. All the experiments were completed in triplicate (*n* = 3), and data are reported as the mean average ± standard deviation.

### 2.7. Drug Delivery Studies

The average dry mass of the microneedle arrays was 0.149 g, and the average water content in the hydrogel microneedles from the swelling/dry weight analysis was 0.140 mL (consequently, the mass fraction of water in the hydrogels was observed to be 51.5%) [42]. Microneedle arrays were incubated in an excess volume of solutions of melatonin, meropenem, or estradiol (each at a concentration of 0.1 g mL^−1^) for 24 h at room temperature (4 patches in 20 mL of solution), after which the microneedles were transferred to a fresh solution for another 24 h to ensure high loading efficiency (the maximum amount of each drug in the microneedle arrays was 14 mg; 98 ± 1% after 2 rounds of loading) [42], which corresponded to a mass fraction of drugs in the aqueous phase of the hydrogels of 10% (14 mg in 0.140 mL [i.e., 140 mg of PBS]), or a mass fraction of drugs in the microneedle arrays of 4.85% (14 mg of drugs in a swollen array of 289 mg).

UV–Vis spectra of samples were recorded using an Agilent Cary 60 UV–Vis spectrophotometer (Agilent Technologies UK Limited, Cheadle, UK), λ_max_ at 200–365 nm, at various times and correlated to a calibration curve to enable the assessment of the cumulative release of melatonin, meropenem, and estradiol at 32 °C into PBS at pH 7.4 from coin-weighted samples. A ten pence coin was attached to the back of the Parafilm^®^-backed melatonin/meropenem/estradiol-loaded microneedle arrays with Parafilm^®^, and placed inside a beaker containing 30 mL of PBS containing a stirrer at 100 strokes/min. Samples from the PBS release medium (3 mL) were extracted at defined time intervals and replaced with an equal volume of fresh PBS. The drug-release kinetics and mechanisms were assessed using the standard literature methods [42]. All the experiments were completed in triplicate (*n* = 3), and data are reported as the mean average ± standard deviation.

The wavelength range for the spectroscopy was set from 200 nm to 360 nm. This range covers the ultraviolet and visible regions of the electromagnetic spectrum, which is suitable for assessing the absorbance of these compounds. The Beer–Lambert law (A = εlc) was employed to correlate absorbance (A) with concentration (c). In this equation, ε represents the molar extinction coefficient, which is specific to each compound. The length of the light path through the solution (l) was measured in centimeters. UV–Vis calibration curves for drug concentration determination with an R^2^ value of close to 1 were utilized for the least-squares linear regression analysis and correlation analysis. The limit of detection (*LoD*) was calculated from Equation (17):

*LoD* = (3.3 × 𝜎) ÷ *S* (17)

where *S* is Slope and *σ* is the SD of the intercept; the *LoD* for melatonin, meropenem, estradiol was found to be 0.081, 0.14, 0.019 ppm. The limit of quantification (*LoQ*) was calculated from Equation (18):

*LoQ* = (10 × 𝜎) ÷ *S* (18)

The *LoQ* for melatonin, meropenem, estradiol was found to be 0.023, 0.008, 0.007 ppm. Zero-order release kinetics describe systems where the rate of drug release remains constant over time. In other words, the amount of drug released per unit of time is consistent, regardless of the amount of drug remaining in the system. Zero-order release kinetics are determined by Equation (19):

C_t_ = C_0_ + K_0_t (19)

where C_t_ is the amount of drug released at time t, C_0_ is the initial concentration of the drug at time t = 0, and K_0_ represents the rate at which the drug is released from the delivery system.

Unless otherwise noted, drugs/buffers, etc., were purchased from Sigma-Aldrich (Gillingham, UK) and used as supplied (the purity of all chemicals was ≥99%).

### 2.8. Statistical Analysis

For each experimental method, a rigorous statistical analysis was performed to ensure the reliability and validity of the obtained results. Measures such as mean, standard deviation, and range, were employed for the assessment of microneedle array fabrication and phantom tissue preparation via Microsoft Excel (Version 2406, Microsoft Corporation, Redmond, WA, USA). Statistical comparisons between different formulations were conducted utilizing t-tests or analysis of variance (ANOVA) using Origin Pro 2022b (OriginLab, Northampton, MA, USA). For the evaluation of microneedle penetration, statistical analyses, ANOVA, or regression analyses were used using Origin Pro 2022b (OriginLab, Northampton, MA, USA). The swelling behavior of phantom tissues was examined by analyzing changes in dimensions or mass over time using Microsoft Excel (Microsoft Corporation, Redmond, WA, USA). Drug delivery studies entail comparisons of drug-release profiles across different formulations or conditions, facilitated by techniques like cumulative release comparisons or kinetic modelling using Origin (OriginLab, Northampton, MA, USA).

## 3. Results

**Figure 1:**
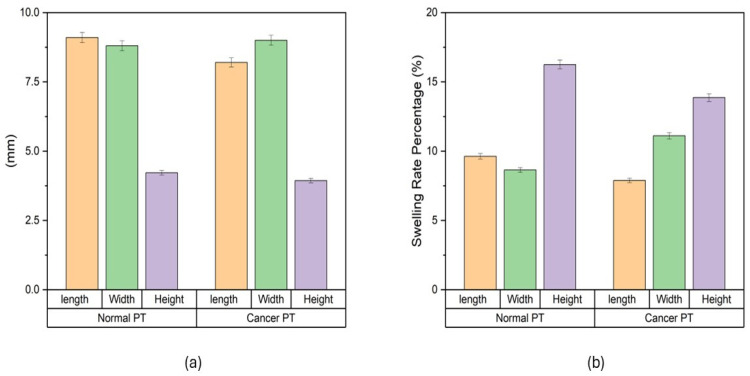
Swelling of phantom tissues in PBS. (**a**) Swelling behavior of phantom tissues for both normal breast tissue and cancerous breast tissue. (**b**) Swelling rate of normal breast phantom tissue and cancerous breast phantom tissue.

The swelling characteristics of both normal and cancer phantom tissues were studied in PBS (Figure 1). Dimensional changes were measured in mm (Figure 1a): for normal phantom tissue, 9.1 ± 0.2 mm for length, 8.8 ± 0.2 mm for width, and 4.2 ± 0.1 mm for height; for cancer phantom tissue, 8.2 ± 0.2 mm for length, 9 ± 0.2 mm for width and 3.9 ± 0.1 mm for height. Swelling rates were measured (Figure 1b). For normal phantom tissue, the average swelling rates were determined to be 9.6% ± 0.2% for length, 8.6% ± 0.2% for width and 15.3% ± 0.3% for height. Concurrently, the cancer phantom tissue exhibited average swelling rates of 7.8% ± 0.2% for length, 11.1% ± 0.2% for width and 13.8% ± 0.3% for height. These values encapsulate the percentage increase in dimensions after the designated time intervals, providing a quantitative representation of the swelling behavior. The average swelling rates highlight nuanced distinctions between normal and cancer phantom tissues. The slightly higher average swelling rates in width and height for normal tissue compared to cancer tissue suggest a more robust and consistent swelling response in the former. Conversely, the greater variability in cancer tissue, as indicated by the standard deviation values, points towards a more heterogeneous swelling behavior.

**Figure 2:**
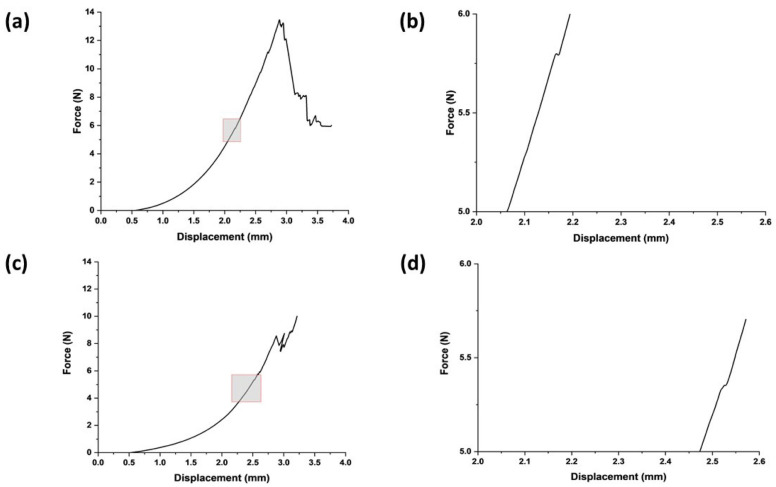
Swollen microneedle penetration study. (**a**) Microneedle insertion into healthy breast phantom tissues. (**b**) Expanded version of the area highlighted in (**a**) focused on microneedle failure in terms of force and displacement for healthy breast phantom tissue. (**c**) Microneedle insertion into cancerous breast phantom tissues. (**d**) Expanded version of the area highlighted in (**c**) focused on microneedle failure in terms of force and displacement for cancerous breast phantom tissue.

The stiffness of the swollen microneedle array was measured to be 0.009 ± 0.001 N/µm, giving an estimated effective modulus for each microneedle in the array of 24.6 MPa (at a displacement distance of 300 µm, the maximum force was 18 N). The microneedles in the swollen state successfully penetrated both the normal breast and cancerous breast phantom tissues (Figure 2); indeed, both were deformed until the first puncture points at approximately 2.5 mm and 2.1 mm, with insertion forces of 5.3 ± 3 N and 5.6 ± 4.1 N, respectively (the differences were not statistically significant). After initial penetration, the microneedles penetrated deeper into the phantom tissue, experiencing a gradual force increase proportional to displacement (N.B., the multiple peaks in the force–displacement curve are due to punctures of phantom tissue layers). The penetration points for both phantom tissues signify a critical threshold for effective microneedle insertion that should elicit minimal discomfort, thus enhancing the potential for efficient and patient-friendly drug delivery systems.

**Figure 3:**
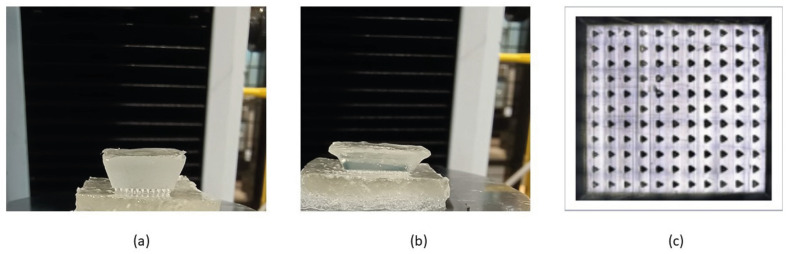
Insertion of swollen microneedle arrays in phantom tissues. (**a**) Microneedle array on the healthy phantom tissue surface. (**b**) Microneedle arrays penetrated the healthy phantom tissue. (**c**) Confocal microscope image of the penetration mark of the 11 × 11 microneedle array on the healthy phantom tissue.

We assessed the number of needles that successfully penetrated the phantom tissues using confocal microscopy to capture the needle marks in the phantom tissues. We observed 11 × 11 array marks on the phantom tissues, confirming that the microneedles had a 100% success in penetrating the phantoms in vitro (Figure 3), suggesting that these microneedles should be capable of penetrating soft tissues in vivo.

**Figure 4:**
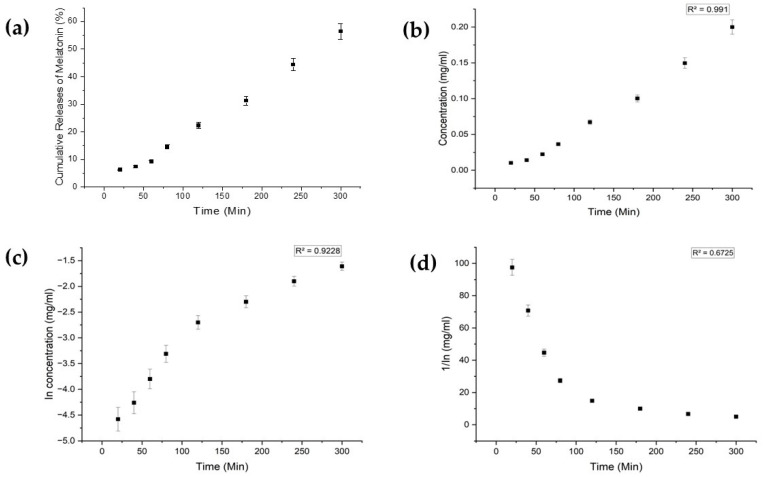
Melatonin release study. (**a**) Cumulative drug release. (**b**) Drug release over 5 h fitted to zero-order kinetics. (**c**) Drug release over 5 h fitted to first-order kinetics. (**d**) Drug release over 5 h fitted to second-order kinetics.

**Figure 5:**
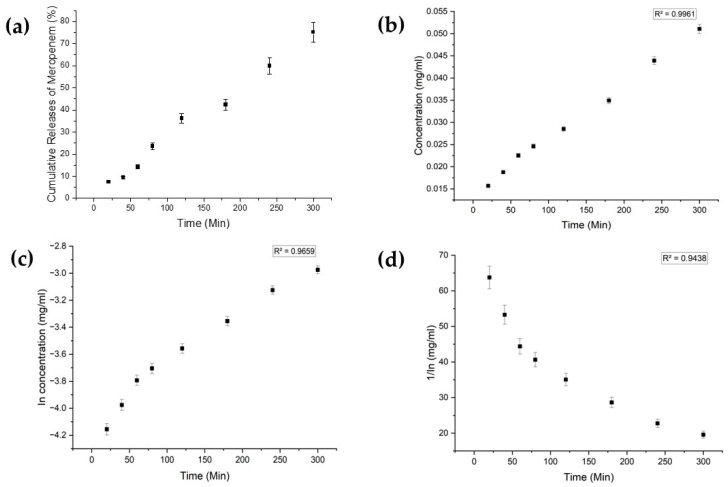
Meropenem release study. (**a**) Cumulative drug release. (**b**) Drug release over 5 h fitted to zero-order kinetics. (**c**) Drug release over 5 h fitted to first-order kinetics. (**d**) Drug release over 5 h fitted to second-order kinetics.

**Figure 6:**
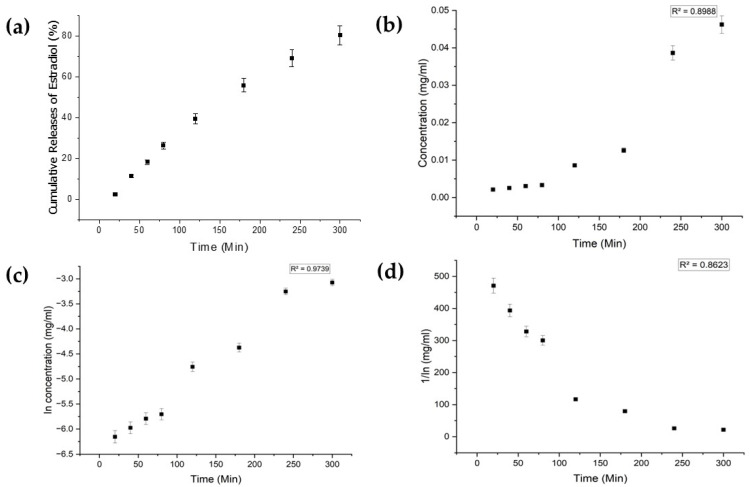
Estradiol release study. (**a**) Cumulative drug release. (**b**) Drug release over 5 h fitted to zero-order kinetics. (**c**) Drug release over 5 h fitted to first-order kinetics. (**d**) Drug release over 5 h fitted to second-order kinetics.

Each drug exhibited distinct characteristics in terms of their limits of detection (LoD) and limits of quantification (LoQ), and their calibration curves had reasonable correlation coefficients (R^2^), as shown in Table A1 (Appendix A). The release profiles of melatonin, meropenem, and estradiol were studied (Figure 4, Figure 5 and Figure 6, respectively). Analysis was undertaken using various empirical release kinetics models, including zero-order, first-order, and second-order models. The suitability of each model was assessed using the regression coefficient method, where a coefficient value (R2) approaching 1 indicated a good fit to the release mechanism. For melatonin and meropenem, the zero-order release plots (Figure 4 and Figure 5) exhibited linear slopes with regression coefficients of 0.991 and 0.996, respectively. Conversely, the release plot for estradiol (Figure 6) demonstrated a linear slope in the first-order model, with a regression coefficient of 0.97.

## 4. Discussion

As noted above, microneedles represent a key enabling technology for the delivery of bioactive molecules transdermally/intradermally with minimal pain/discomfort, which is important for enhancing patient compliance [51,52,53,54,55]. Here, we utilized medical model tissues (“phantom tissues”) that replicated the characteristics of healthy/unhealthy tissues, specifically healthy and cancerous breast tissues. The mechanical properties of the phantom tissues were analogous to values reported for sections of healthy tissues and cancerous breast tissues measured ex vivo in the literature [14,45,46].

The length of the pHEMA microneedles in this study was ≈238 ± 97 μm [42], designed with dimensions such that their insertion should elicit minimal discomfort when inserted in tissues [54,55]. The pHEMA microneedles successfully penetrated both the normal breast and cancerous breast phantom tissues with effective microneedle insertion at forces which should elicit minimal discomfort, thus enhancing the potential for efficient and patient-friendly drug delivery systems.

We have previously employed these pHEMA microneedles for the delivery of metformin in vitro [42], and here we demonstrate their efficacy in delivering other bioactive molecules in vitro, specifically the hormones estradiol and melatonin, and a broad-spectrum antibiotic meropenem, exhibiting first-order release kinetics for estradiol and zero-order release kinetics for melatonin and meropenem, suggesting their broad applicability with low-molecular-weight drugs.

Using such phantom tissues as models for soft tissues helps bridge the gap between in vitro and in vivo studies, providing a reproducible environment for investigations into microneedle–tissue interactions and opportunities to optimize insertion parameters to ensure patient comfort/compliance. Long-term studies involving systematic force measurements should facilitate the establishment of a comprehensive framework for designing microneedle systems with optimal tissue penetration capabilities; likewise, tuning the polymer hydrogel chemistry would offer opportunities for tuning the physicochemical properties of the gels and the release profiles of the drugs in polymer matrices.

## 5. Conclusions

The inexpensive pHEMA hydrogel microneedle arrays described herein are simple to prepare and to load with drugs, as exemplified with estradiol, melatonin and meropenem, which display a variety of biological activities. The microneedles are robust enough to penetrate normal breast and cancerous breast phantom tissues with effective microneedle insertion at forces that should elicit minimal discomfort if applied to humans. The simple manufacture of such microneedles offers opportunities to integrate them in biomedical devices used for the transdermal/intradermal delivery of drugs.

## Data Availability

The data that support the findings of this study are available from the corresponding author upon reasonable request.

## Appendix A

**Table A1:** Drug detection parameters.

Drug	Melatonin	Meropenem	Estradiol
Wavelength of λ_max_ (nm)	320	310	281
LoD (ppm)	0.023	0.008	0.007
LoQ (ppm)	0.081	0.14	0.019
Correlation coefficient (R^2^)	0.92	0.96	0.97
